# Interactive effects of high n-3 PUFA intake and cyclic heat stress under two dietary antioxidant levels in broiler chickens

**DOI:** 10.3389/fphys.2025.1594095

**Published:** 2025-04-24

**Authors:** Vida Rezar, Manca Pečjak Pal, Alenka Levart, Alenka Nemec Svete, Tatjana Pirman, Janez Salobir, Jakob Leskovec

**Affiliations:** ^1^ Department of Animal Science, Biotechnical Faculty, University of Ljubljana, Ljubljana, Slovenia; ^2^ Clinic for Surgery and Small Animal Medicine, Veterinary Faculty, University of Ljubljana, Ljubljana, Slovenia; ^3^ Animal Nutrition, Institute for Food and Agricultural Research and Technology (IRTA), Tarragona, Spain

**Keywords:** cyclic heat stress, n-3 PUFA, oxidative stress, selenium, vitamin C, vitamin E

## Abstract

The study examined the combined effects of a diet high in n-3 PUFAs and cyclic heat stress (HS) considering two levels of dietary antioxidants. A total of 192 one-day-old male Ross 308 broilers were divided into eight groups in a 2 × 2 × 2 factorial design: thermoneutral (TN) or heat-stressed (34°C ± 1°C for 7 h/day from day 22) × fed a diet low in antioxidants (NRC group) or high in antioxidants (HAOX group; supplemented with a mixture of 200 IU/kg vitamin E, 250 mg/kg vitamin C and 0.15 mg/kg selenium) and supplemented or not with 5% linseed oil, forming the NRC N-3 and HAOX N-3 groups. High intake of n-3 PUFAs increased plasma F2-isoprostane and malondialdehyde (MDA) levels and superoxide dismutase (SOD) activity. Cyclic HS decreased final body weight (BW) and average daily feed intake (ADFI) and increased DNA damage and serum corticosterone (CORT) levels. In addition, the changes in blood biochemistry indicated that the broilers were undergoing respiratory alkalosis. Interactions between n-3 PUFAs and HS were observed in liver MDA levels, plasma γ-tocopherol levels and serum alkaline phosphatase (AP) activity. Antioxidants increased blood levels of α-tocopherol, vitamin C, lipid- and water-soluble antioxidants and glutathione peroxidase (GPx) activity, while decreasing MDA, F2-isoprostane, CORT and AP levels. High intake of n-3 PUFA in combination with cyclic HS had negative effects on the health status of the broilers, which were alleviated by additional antioxidant supplementation.

## 1 Introduction

Poultry production is often associated with various external stressors that lead to an overproduction of free radicals and consequently to oxidative stress. In broiler diets, energy rich components are required to increase productivity, which is achieved by the inclusion of various fats and oils. Recently, there has been growing interest in the production of functional foods that provide additional health benefits to consumers. Supplementing broiler diets with plant oils rich in n-3 polyunsaturated fatty acids (PUFAs) is one of the widely accepted methods to improve the nutritional value of chicken meat (Long et al., 2020). However, n-3 PUFAs are highly susceptible to oxidation, leading to the formation of lipid peroxides and thus to an increased susceptibility to oxidative deterioration (Voljč et al., 2011; Konieczka et al., 2018). Previous studies have confirmed that higher intake of n-3 PUFA-rich oils increases lipid peroxidation, as evidenced by increased malondialdehyde (MDA) levels in blood and tissues, increases DNA damage in blood lymphocytes and epithelial cells, and decreases antioxidant capacity, such as α-tocopherol concentrations in plasma and liver of broilers (Voljč et al., 2011; Tomažin et al., 2014; Long et al., 2020). On the other hand, modern broiler genotypes with improved growth rate, feed efficiency, and muscle deposition are more susceptible to elevated ambient temperatures, leading to heat stress (HS), due to their greater metabolic activity and increased heat production (Nawab et al., 2018). Birds exposed to HS release excessive heat into the environment through panting, which leads to respiratory alkalosis, impaired performance, and higher mortality (Habibian et al., 2016; Calik et al., 2022b). Exposure to HS has been associated with DNA damage, as indicated by higher plasma 8-hydroxy-2′deoxyguanosine (8-OHdG), increased blood corticosterone (CORT) levels, heat shock protein 70 (Hsp70) expression, and enhanced lipid peroxidation in blood and tissues as indicated by higher MDA concentrations (Altan et al., 2003; Najafi et al., 2015; Beyzi et al., 2020; Pečjak et al., 2022). In addition, Yang et al. (2010) reported that exposure of broilers to acute HS induced higher reactive oxygen species (ROS) production, resulting in lipid peroxidation of liver and serum, reduction of mitochondrial respiratory chain, and upregulation of antioxidant enzyme activity, including glutathione peroxidase (GPx), catalase, and superoxide dismutase (SOD). In our previous studies, we have already investigated the individual effects of high n-3 PUFA intake and cyclic HS (Voljč et al., 2011; Leskovec et al., 2018; Pečjak et al., 2022) and confirmed their association with the induction of oxidative stress. However, to our knowledge, the interaction between these two stressors on oxidative stress and antioxidative status of broilers has not yet been studied. In addition, only a few studies have investigated the combined effect of different stressors, particularly high stocking density and HS, on broiler performance and antioxidative status. In these studies, a significant interaction between temperature and stocking density was found for CORT levels (Najafi et al., 2015), while performance parameters were negatively affected when broilers were exposed to either HS or high stocking density as an individual stressor (Goo et al., 2019). Various environmental and nutritional interventions have been proposed to mitigate the negative effects of oxidative stress caused by external stressors and to enhance the efficiency of antioxidant defense mechanisms. Among the commonly used nutritional strategies, supplementation with vitamin E, vitamin C and selenium (Se) has shown promising results in attenuating the adverse effects of HS and high intake of n-3 PUFAs (Attia et al., 2017; Leskovec et al., 2018; Calik et al., 2022a). Vitamins E, C and Se play an important role in the antioxidant defense system and act synergistically to increase antioxidant activity and decrease oxidative stress by reducing free radical production when supplemented together. This synergistic effect has been confirmed in previous studies, reporting positive interactions between vitamins E and C (Sahin et al., 2002; Attia et al., 2017) and between vitamin E and Se (Ghazi Harsini et al., 2012; Habibian et al., 2016; Kumbhar et al., 2018). The present study had two main objectives. First, we aimed to investigate the interaction between high dietary n-3 PUFA intake and cyclic HS on oxidative stress and antioxidant defense parameters in broiler chickens. We hypothesized that both stressors would have negative effects, with the combined effect being more profound. Second, under conditions of high oxidative load (n-3 PUFA and HS), we sought to determine the benefits of supranutritional supplementation with vitamins E, C and Se to attenuate oxidative stress. Conversely, under stress-free conditions, antioxidant supplementation may not be necessary as current recommendations already consider the basic antioxidant requirements.

## 2 Materials and methods

### 2.1 Animal ethics

All experimental procedures applied in this study were performed according to current legislation on animal experimentation in Slovenia, which comply with the EU regulations regarding research on experimental animals. The protocol was approved by the Animal Ethics Committee of the Veterinary Administration of the Republic of Slovenia (No. U34401-5/2021/4). The nutritional study was performed in the research facility of the Department of Animal Science, Biotechnical Faculty, University of Ljubljana, Slovenia. The present study was part of a larger experiment, which previous results regarding gut fermentation, mucosal morphology, meat quality and oxidative stability of broiler breast meat have already been published (Rezar et al., 2023; Pal et al., 2024).

### 2.2 Broiler chickens, housing, and dietary treatments

**FIGURE 1:**
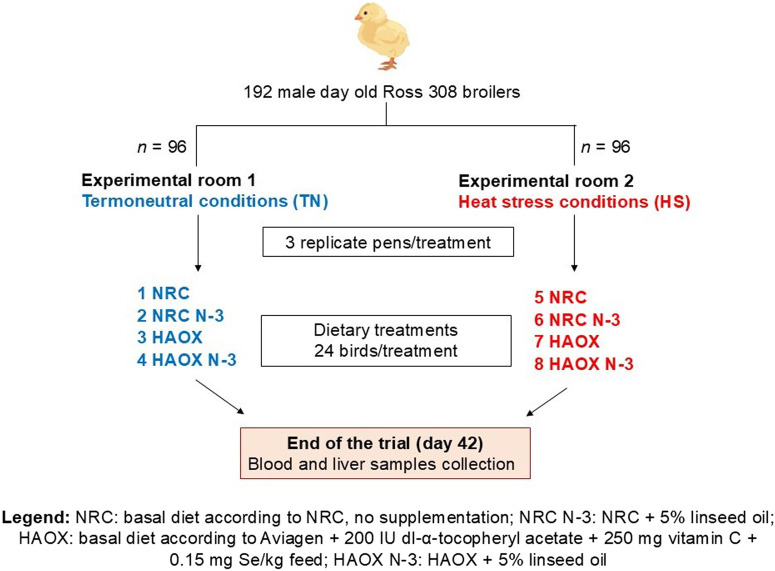
Schematic view of the experimental design.

A total of 192 one-day-old male Ross 308 broiler chickens were randomly assigned to 24 deep litter pens using a 2 × 2 × 2 factorial design in the randomized complete block design. The trial induced four dietary treatments (2 × 2 diets) and two environmental conditions: thermoneutral (TN) and heat stress (HS). Birds were divided evenly between two separate experimental rooms (TN or HS; 96 birds per room) in the same experimental facility. Each experimental group consisted of three replicate pens, each measuring 0.95 m × 1.26 m (1.20 m^2^), with wood shavings as litter and equipped with a plastic feeder and five nipple drinkers. Feed and water were provided *ad libitum* throughout the study period. The broilers were reared under a standard lighting program: 23 h of light and 1 h of darkness in the first week, followed by 18 h of light and 6 h of darkness from day 8 until the end of the trial. Each bird was individually marked and body weight (BW) was recorded weekly and on the day of slaughter. Average daily feed intake (ADFI), body weight gain (BWG), and feed conversion ratio (FCR) were recorded to determine growth performance. The experimental diets were administered in three phases: a starter diet from day 1 to 10, a grower diet from day 11 to 24 and a finisher diet from day 25 to day 42. The experimental diets were formulated either based on the NRC minimal nutrient requirements (NRC, 1994) (low antioxidants) or according to Aviagen’s recommendations for Ross 308 broilers (Aviagen, 2019), supplemented with 200 IU vitamin E, 250 mg vitamin C and 0.15 mg Se/kg feed (high antioxidant content). Additionally, in both experimental diets the primary fat source (mixture of animal fats and plant oils) was replaced with 5% cold-pressed linseed oil to obtain PUFA enriched experimental diets. This design resulted in four dietary treatments: a basal diet following NRC recommendations without additional supplementation (NRC group), a basal diet following Aviagen recommendations + 200 IU/kg vitamin E + 250 mg/kg vitamin C + 0.15 mg/kg Se (HAOX group), NRC + 5% linseed oil (NRC N-3 group) and HAOX + 5% linseed oil (HAOX N-3 group). The components and the calculated energy and nutrient contents of the starter, grower and finisher diet formulations are listed in Table 1. The schematic design of the experimental procedure is shown in Figure 1. Samples of the finisher diets were collected to determine the proximate and mineral content, calculated dietary electrolyte balance (DEB), composition of α- and γ-tocopherol, the MDA content, the antioxidant capacity of water-soluble (ACW) and lipid-soluble (ACL) antioxidants, and fatty acids (FA) composition (Table 2).

**TABLE 1:** Composition and calculated nutrient content of the experimental diets fed to broiler chickens during the starter (1–10 days), grower (11–24 days) and finisher (25–42 days) period.

	Starter	Grower	Finisher
Composition of Experimental Diets^a^			
Maize, g/kg	309.8	379.9	526.8
Wheat, g/kg	140.0	130.0	40.0
Wheat flour, g/kg	30.0	15.0	0.0
Soya meal, g/kg	332.2	280.0	240.0
Corn gluten meal, g/kg	86.0	93.0	94.1
Mixture of animal fats and plant oils or linseed oil, g/kg	54.0	57.2	57.2
Salt, g/kg	4.91	4.95	5.05
Monocalcium phosphate, g/kg	17.10	15.80	14.50
Limestone, g/kg	14.50	13.40	12.20
L-lysine-HCl, g/kg	3.50	3.40	3.30
DL-methionine, g/kg	2.20	1.80	1.50
L-threonine, g/kg	0.80	0.50	0.30
Mineral-vitamin mix, g/kg^b^ ^,^ ^c^	5.00	5.00	5.00
Calculated Energy and Nutrient Contents			
Metabolizable energy, MJ/kg	12.42	12.82	13.20
Crude protein, g/kg	246.0	228.0	208.0
Lysine, g/kg	12.84	11.48	10.28
Methionine, g/kg	5.97	5.42	4.95
Calcium, g/kg	9.56	8.75	7.91
Available phosphorus, g/kg	4.80	4.42	4.03

Composition and nutrient content of the experimental diets as in Rezar et al. (2023) and Pal et al. (2024).

^a^ All diets contain coccidiostat Maxiban® G160 (Elanco Products Co., hook, Hampshire, United Kingdom).

^b^ Calculated to meet the mineral and vitamin requirements for NRC, finisher diets and Aviagen finisher diets for Ross 308 broilers and provided per kilogram of the diet. Due to the sufficient amount of l-α-tocopherol acetate in the mixture of animal fats and plant oils and linseed oil, no additional vitamin E was added to the NRC, finisher diets.

^c^ In the HAOX, and HAOX N-3 experimental diets, the following antioxidants were used for supplementation: as a source of vitamin E (dl-α-tocopheryl acetate), Rovimix E50 (DSM, heerlen, Netherlands); as a source of vitamin C, Rovimix Stay-C35 (DSM, heerlen, Netherlands); and as an organic Se source (mainly L (+)-selenomethionine), SelSaf 3000 (Lesaffre, Marcq en Baroeul, France).

**TABLE 2:** Proximate composition, concentration of minerals, calculated dietary electrolyte balance (DEB), content of α-, and γ-tocopherol, antioxidant capacity of water- (ACW) and lipid- (ACL) soluble compounds, MDA and the FA composition of the finisher experimental diets.

Component	Dietary treatments^a^			
	NRC	NRC N-3	HAOX	HAOX N-3
Dry matter, g/kg	899.3	898.5	897.6	897.5
Crude protein, g/kg	215.0	213.7	214.1	212.6
Ether extract, g/kg	84.39	86.70	83.82	84.07
Crude ash, g/kg	55.04	56.65	58.93	54.83
Crude fiber, g/kg	68.40	71.19	68.81	75.14
Nitrogen free extract, g/kg	476.4	470.2	471.9	470.8
K, g/kg	8.76	8.57	8.84	9.02
Ca, g/kg	7.55	7.87	8.41	7.24
P, g/kg	5.91	5.95	6.25	5.94
Mg, g/kg	1.64	1.69	1.72	1.69
Na, g/kg	2.51	2.60	2.85	2.64
Fe, mg/kg	283.9	217.9	164.1	147.7
Zn, mg/kg	74.59	79.29	151.8	141.2
Mn, mg/kg	96.31	105.3	187.1	148.8
Cu, mg/kg	15.47	19.75	21.64	22.97
Se, mg/kg	0.175	0.147	0.387	0.351
Calculated DEB, mEq/kg	214.8	214.0	232.0	227.0
Tocopherol Isomers				
α-tocopherol, mg/kg	12.15	8.87	250.2	232.2
γ-tocopherol, mg/kg	26.76	43.37	27.23	45.09
MDA, ACW and ACL				
MDA, nmol/g	3.81	7.53	3.35	4.40
ACW, µmol/g	3.37	2.62	2.95	2.51
ACL, µmol/g	0.34	0.25	0.27	0.27
Fatty Acid Composition^c^, g of fatty acids/100 g of fatty acids				
C16:0	22.36	8.99	22.09	8.95
C18:0	10.97	3.66	10.66	3.61
C18:2 n-6	26.53	28.36	27.53	28.52
C18:3 n-3	2.08	37.84	2.09	37.77
∑ SFA	37.02	13.40	36.34	13.30
∑ MUFA	33.99	20.41	33.65	20.40
∑ PUFA	28.97	66.20	29.98	66.29
∑ n-6 PUFA	26.75	28.36	27.74	28.52
∑ n-3 PUFA	2.14	37.84	2.16	37.77
n-6/n-3 PUFA	12.53	0.75	12.87	0.76

The content of Table 2 as in Rezar et al. (2023) and Pal et al. (2024). MDA, malondialdehyde; ACW, antioxidant capacity of water-soluble compounds; ACL, antioxidant capacity of lipid-soluble compounds; ∑ = sum of isomers; SFA, saturated fatty acid; MUFA, monounsaturated fatty acid; PUFA, polyunsaturated fatty acid.

^a^ NRC: basal diet according to NRC, no supplementation; NRC N-3: NRC + 5% linseed oil; HAOX: basal diet according to Aviagen + 200 IU, dL-α-tocopheryl acetate + 250 mg vitamin C + 0.15 mg Se/kg feed; HAOX N-3: HAOX + 5% linseed oil.

^b^ All values are means of two analyses per measured sample.

^c^ Only prevalent and dietary important fatty acids are listed.

### 2.3 Temperature treatments

**FIGURE 2:**
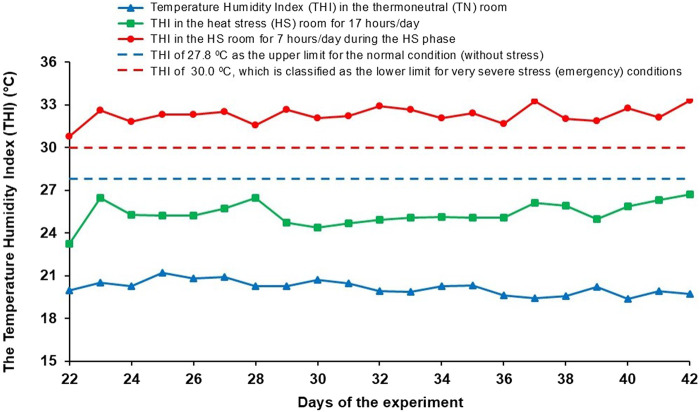
Daily temperature humidity index (THI) in the TN room and HS room when exposed to HS (7 h/day), and as a rest of the day (17 h/day).

The chickens in the TN groups were reared at controlled ambient temperature and humidity throughout the trial, according to the recommendations for Ross 308 broilers (Aviagen, 2018). The same environmental conditions were maintained in the HS room as in the TN room until day 22. To trigger the cyclic HS, a modified temperature regime was introduced from day 22 until the end of the trial. During this period, the chickens underwent a daily cycle consisting of 12 h at 24°C ± 0.5°C, a 2-h warm-up phase in which the temperature gradually increased from 24°C ± 0.5°C to 34°C ± 1°C, 7 h at 34°C ± 1°C (HS phase) and a 3-h cool-down phase in which the temperature decreased from 34°C ± 1°C to 24°C ± 0.5°C. The relative humidity was monitored daily and allowed to fluctuate, but never to fall below 45%. To determine the effects of the thermal environment on the thermoregulatory status of broilers and thus determine the thermal comfort of the chickens, especially during HS, the temperature-humidity index (THI) was calculated daily for the THI for broilers (Figure 2).

### 2.4 Experimental procedure and sample collection

A 2 days before the end of the experiment, 9 birds per group (3 birds per pen) and per room were randomly selected for blood sampling. Two milliliters of blood were collected from the wing vein in plastic tubes containing the anticoagulant K2 EDTA (No. 454086, Vacuette, Greiner Bio-One, Cassina de Pecchi, Italy) to determine DNA fragmentation in the blood lymphocytes. Samples were stored in a dark and cool place immediately after collection and cell isolation was performed within 1 h of collection. On day 42, 4 birds per pen (12 birds per group and room) were randomly selected and weighed. Blood gas analysis was performed to determine blood chemistry, hematology and acid-base balance. For this purpose, 1 mL of blood was collected from the wing vein of each broiler before slaughter in plastic tubes containing lithium heparin (Vacuette, Greiner Bio-One, Cassina de Pecchi, Italy). Immediately after collection, the samples were analyzed on a CG8+ cartridge with a portable blood gas analyzer (i-STAT Alinity, Abbott Point of Care Inc., Illinois, United States). Immediately after the blood analysis, the broilers were sacrificed by cervical dislocation and exsanguination. Blood samples were collected for further analysis. The blood samples for the determination of F2-isoprostanes, MDA, Hsp70, α- and γ-tocopherol and vitamin C in plasma were collected in plastic tubes containing K2 EDTA (Vacuette, Greiner Bio-One, Cassina de Pecchi, Italy) and separated by centrifugation (1,000 × g at 4°C for 10 min). Vitamin C in plasma was stabilized by adding 250 μL of 10% m-phosphoric acid to 250 μL of plasma before storage. For the analysis of 8-OHdG, CORT, the biochemical profiles (aspartate aminotransferase (AST), alanine aminotransferase (ALT), gamma-glutamyl transferase (GGT), alkaline phosphatase (AP), sodium, potassium and chloride), ACL and ACW in serum, blood samples were collected in plastic serum separator tubes without anticoagulant (Vacuette, Greiner Bio-One, Cassina de Pecchi, Italy) and left at room temperature to allow the blood to clot. To obtain the serum, the tubes were centrifugated at 2,000 × g at 4°C for 10 min. For the determination of GPx and SOD activities, whole blood samples were collected in tubes containing lithium heparin. Liver samples were collected and stored in polypropylene plastic bags for the determination of MDA and tocopherols. All collected samples were stored at −80°C until further analysis.

### 2.5 Chemical analyses

The fatty acids in the experimental diets were analyzed by gas chromatography after the *in situ* transmethylation, as described by Leskovec et al. (2018). Serum electrolyte concentrations (sodium, potassium and chloride) were determined using an electrolyte analyzer (ILyte, Instrumentation Laboratory, Lexington, Massachusetts, United States). The Comet assay was performed to determine the percentage of head and tail DNA and olive tail moment (OTM), calculated as the product of the total DNA fraction in the tail and its length, according to the method of Voljč et al. (2011), except different software (Komet, version 7.0.1 08, Andor Technology Ltd., Belfast, United Kingdom) and a fluorescent nucleic acid dye (GelRed, Biotium, CA, United States) were used. Commercial ELISA kits were used to analyze serum 8-OHdG (No. ADI-EKS-350, Enzo Life Science, Farmingdale, NY, United States), serum CORT (No. 501320, Cayman Chemical, Ann Arbor, MI, United States) and plasma F2-isoprostanes (iPF2α- III) (No. 516351, Cayman Chemical, Ann Arbor, MI, United States) and Hsp70 (Wuhan Fine Biotech Co. (No. ECH00723)). The MDA content in feed, liver and plasma was measured by HPLC (Agilent, Santa Clara, United States) according to the method of Wong et al. (1987) with some modifications described by Voljč et al. (2011). Liver enzymes (AST, ALT, GGT, and AP) in broiler serum were determined using an automated biochemical analyzer (RX Daytona, Randox, Crumlin, United Kingdom). Concentrations of tocopherols in feed, liver and plasma samples as well as plasma vitamin C were determined by HPLC (Agilent, Santa Clara, United States) according to the protocol described by Leskovec et al. (2018). The ACW and ACL in feed and serum samples were analyzed using commercial PhotoChem kits (Analytik Jena, Jena, Germany). The activities of SOD and GPx in whole blood were determined spectrophotometrically with an automated biochemical analyzer RX Daytona (Randox Laboratories, Crumlin, United Kingdom) using commercially available Ransod and Ransel kits (Randox Laboratories, Crumlin, United Kingdom), as the procedure is described in detail in the studies by Leskovec et al. (2018) and Pirman et al. (2021).

### 2.6 Statistical analysis

The statistical analysis of the data was performed using the MIXED procedure of the SAS software (version 9.4, SAS Institute Inc. Cary, NC, United States). In the statistical model, environmental conditions (E), dietary fat treatments (F), antioxidant supplementation (A) and their interactions (E × F, E × A, F × A and E × F × A) were determined as fixed effects and replication pen as a random effect. For FI and FCR, a three-way ANOVA was performed using E, F, A and their interactions as fixed effects. If the interactions between the fixed effects were significant, comparisons were made within each experimental variable. Furthermore, for the results of the Comet assay, the measured values of the cells for each bird within an experimental unit were considered as a random effect. Least square means (LSMs) were estimated by applying the LSMEANS statement in the model. Differences between LSMs were determined by a Tukey-Kramer multiple comparison test. Dispersion was expressed as the standard error of the mean (SEM). Statistical significance of the results was considered at p < 0.05.

## 3 Results

### 3.1 Growth performance

**FIGURE 3:**
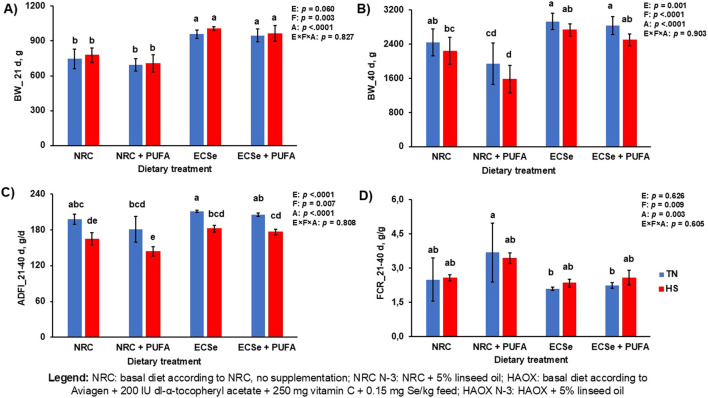
Performance parameters in broilers during the experimental period. **(A)** Body weight (BW) at day 21; **(B)** Body weight (BW) at day 40; **(C)** Average daily feed intake (ADFI) during the period from 21 to 40 days; **(D)** Feed conversion ratio (FCR) during the period from 21 to 40 days. Mean values are based on 8 birds per replicate and three replicates per dietary treatment, n = 12. ADFI and FCR are expressed as an average amount per experimental group, since broilers were housed in pens (3 pens per group). Different letters above the bars indicate significant differences between the experimental groups (p < 0.05).

Performance was generally affected by all parameters included. At day 21, the inclusion of PUFA in the diet lowered BW, while high antioxidant supplementation in the HAOX groups increased it. At day 40, HS, PUFAs and low levels of antioxidants (NRC N-3 group) lowered BW and ADFI compared to TN conditions without PUFA and high antioxidants levels (HAOX group) (Figure 3). A significant interaction between dietary fat and antioxidant supplementation was observed for BW at day 40 (p = 0.010), with the lowest BW recorded when low antioxidants and high PUFAs were administered (Supplementary Table S1). FCR was negatively affected by high PUFA levels and low levels of antioxidants, with the addition of PUFAs and the NRC groups caused worse FCR than group without PUFA and HAOX groups (Figure 3).

### 3.2 Blood chemistry parameters and serum electrolytes

In the present trial, the inclusion of n-3 PUFAs to the experimental broiler diet caused an increase in partial pressure of oxygen (pO_2_) and glucose and a decrease in hematocrit (Hct), hemoglobin (HGB) and ionized calcium (iCa) compared to groups fed conventional fat. HS generally affected all blood chemistry parameters in a manner indicative of respiratory alkalosis. In addition, HS caused an increase in Na+ and Cl- levels and a decrease in iCa levels compared to birds reared under TN conditions. High antioxidant levels resulted in lower pO_2_, higher pH, Na+ and iCa compared to NRC groups (Table 3). A significant interaction between environment and antioxidant supplementation was observed for base excess of the extracellular fluid (BEecf) (p = 0.049) and Na+ (p < 0.0001). The combination of HS and low antioxidants levels reduced BEecf, while high antioxidants levels increased Na+ levels under HS. In addition, an interaction between dietary fat and antioxidant supplementation showed that a diet high in n-3 PUFA and low in antioxidants increased blood glucose levels (p = 0.021) (Supplementary Table S2).

**TABLE 3:** Blood chemistry parameters in venous blood and serum electrolyte (Na+, K+, and Cl-) levels of broilers fed different experimental diets and reared under different environmental conditions at the end of the trial.

Item^a^/Diet^b^	Environment								p-Value					
	Thermoneutral (TN)				Heat stress (HS)				SEM	Environment (E)	Fat (F)	Antioxidants (A)	E × F	E × F × A
	NRC	NRC N-3	HAOX	HAOX N-3	NRC	NRC N-3	HAOX	HAOX N-3						
Blood Chemistry Parameters														
TCO_2_, mmol/L	26.42^a^	26.42^a^	26.08^a^	26.17^a^	23.00^ab^	21.75^b^	23.84^ab^	23.01^ab^	0.800	<0.0001	0.384	0.506	0.346	0.881
pCO_2_, mmHg	36.48^a^	37.08^a^	34.52^a^	33.76^ab^	27.23^bc^	27.41^bc^	25.73^c^	25.43^c^	1.541	<0.0001	0.946	0.051	0.994	0.845
pO_2_, mmHg	48.19^ab^	50.45^ab^	46.82^b^	50.33^ab^	48.62^ab^	51.37^a^	46.91^ab^	47.76^ab^	1.005	0.704	0.002	0.025	0.463	0.290
sO_2_, %	85.82	85.92	85.67	88.18	89.43	89.17	88.58	89.17	0.970	0.0002	0.293	0.649	0.411	0.573
pH	7.455^bc^	7.442^c^	7.473^bc^	7.480^bc^	7.518^ab^	7.499a^bc^	7.560^a^	7.554^a^	0.016	<0.0001	0.510	0.001	0.661	0.870
HCO_3_^−^, mmol/L	25.30^a^	25.23^a^	25.01^a^	25.09^a^	22.09^ab^	20.92^b^	23.14^ab^	22.31^ab^	0.779	<0.0001	0.369	0.365	0.365	0.936
BEecf, mmol/L	1.167^a^	1.083^a^	1.500^a^	0.637^ab^	−0.583^ab^	−2.416^b^	1.417^a^	−0.106^ab^	0.771	0.008	0.056	0.062	0.279	0.623
Glu, mg/dL	230.2^b^	254.9^b^	228.7^b^	233.7^b^	236.6^b^	320.8^a^	255.4^b^	259.2^ab^	15.22	0.005	0.007	0.127	0.173	0.156
Hematology														
Hct, % PVC	22.25	19.75	21.50	21.08	20.58	20.42	21.42	20.00	0.704	0.280	0.027	0.617	0.505	0.099
HGB, g/dL	7.567	6.717	7.300	7.167	6.992	6.950	7.283	6.808	0.236	0.286	0.027	0.618	0.486	0.089
Blood Electrolytes														
Na+ in serum, mmol/L	149.3^bc^	148.7^c^	149.1^bc^	148.0^c^	149.5^abc^	146.7^c^	152.3^ab^	152.8^a^	0.743	0.005	0.066	0.0003	0.768	0.075
K+ in serum, mmol/L	5.768	5.977	5.602	4.892	5.785	5.687	5.312	5.242	0.307	0.798	0.421	0.011	0.688	0.256
Cl- in serum, mmol/L	108.1	107.7	108.4	107.0	110.8	106.9	111.1	111.9	1.228	0.008	0.150	0.159	0.685	0.110
iCa in VB, mmol/L	1.336^ab^	1.238^b^	1.421^a^	1.387^a^	1.245^b^	0.948^c^	1.368^ab^	1.316^ab^	0.028	<0.0001	<0.0001	<0.0001	0.011	0.033

^a^ Abbreviations: TCO_2_, Total CO_2_ concentration; pCO_2_, Partial pressure of CO_2_; pO_2_, Partial pressure of O_2_; sO_2_, oxygen saturation; HCO3-, bicarbonate; BEecf, Base excess of the extracellular fluid; Glu, Blood glucose; Hct, Hematocrit; HGB, hemoglobin; iCa, Ionized calcium; VB, venous blood.

^b^ Nomenclature of experimental groups as in Table 2.

^c^ Different superscript letters within the row show significant differences (p < 0.05). Mean values are based on 4 birds per replicate and three replicates per dietary treatment, n = 12.

### 3.3 Markers of oxidative stress in blood

Inclusion of n-3 PUFAs in the diet caused in a decrease in serum 8-OHdG and AST and an increase in plasma F2-isoprostanes, plasma and liver MDA, and serum GGT and AP compared to groups without n-3 PUFAs. Cyclic HS caused markers of DNA damage to elevate as measured by tail DNA, OTM, serum 8-OHdG, corticosterone and GGT compared to TN conditions. Inclusion of high levels of antioxidants reduced plasma and liver MDA, serum corticosterone and AP, while AST levels increased compared to the NRC groups with low antioxidant levels (Table 4). A significant interaction between dietary fat and antioxidant supplementation was observed in plasma MDA levels (p = <0.0001), with high PUFA levels without antioxidant supplementation showing increased lipid peroxidation (Supplementary Table S3).

**TABLE 4:** Parameters of oxidative stress, corticosterone (CORT) and heat shock protein 70 (Hsp70) levels, and activities of liver enzymes measured in broiler plasma, liver and serum.

Item^a^/Diet^b^	Environment								p-Value					
	Thermoneutral (TN)				Heat stress (HS)				SEM	Environment (E)	Fat (F)	Antioxidants (A)	E × F	E × F × A
	NRC	NRC N-3	HAOX	HAOX N-3	NRC	NRC N-3	HAOX	HAOX N-3						
DNA Damage														
Tail DNA, %	19.59^bc^	19.75^abc^	19.35^c^	19.47^c^	20.81^a^	20.76^ab^	20.58^abc^	20.54^abc^	0.267	<0.0001	0.808	0.212	0.618	0.949
OTM	4.301^bc^	4.198^c^	4.142^c^	4.365^bc^	5.012^a^	4.995^a^	4.950^a^	4.669^ab^	0.096	<0.0001	0.526	0.179	0.140	0.037
Serum 8-OHdG, ng/mL	27.30^bc^	22.34^c^	33.81^ab^	29.94^bc^	32.54^ab^	29.79^bc^	40.05^a^	28.39^bc^	2.014	0.004	0.0002	0.001	0.348	0.094
Lipid Oxidation														
Plasma F2-isoprostanes, pg/mL	1280.5^ab^	821.7^bc^	972.0^abc^	688.4^c^	1418.1^a^	823.4^bc^	961.7^abc^	665.7^c^	124.7	0.753	<0.0001	0.003	0.661	0.714
Plasma MDA, nmol/mL	0.118^b^	0.224^a^	0.127^b^	0.165^b^	0.143^b^	0.253^a^	0.124^b^	0.161^b^	0.012	0.181	<0.0001	<0.0001	0.898	0.880
Liver MDA, nmol/g	1.386^bc^	1.861^b^	0.574^c^	0.995^c^	1.027^c^	2.654^a^	0.619^c^	1.113^bc^	0.158	0.205	<0.0001	<0.0001	0.010	0.024
Stress Indicator														
Serum Corticosterone, pg/mL	2270.1^ab^	1691.9^ab^	1183.9^b^	657.5^b^	4129.6^a^	4474.9^a^	1992.6^ab^	2565.9^ab^	627.0	0.0002	0.918	0.001	0.268	0.923
Plasma Hsp70, ng/mL	3.741	3.662	3.517	3.645	3.638	3.858	3.897	3.596	0.145	0.323	0.937	0.568	0.762	0.091
Liver Enzymes														
Serum AST, U/L	493.9^abc^	321.2^c^	588.7^ab^	523.5^abc^	444.9^bc^	382.7^bc^	686.2^a^	529.7^abc^	55.55	0.458	0.005	<0.0001	0.902	0.199
Serum ALT, U/L	1.612	0.932	1.607	1.384	1.239	1.436	1.776	1.763	0.276	0.321	0.293	0.058	0.114	0.330
Serum GGT, U/L	17.46^b^	18.48^ab^	18.81^ab^	18.42^ab^	17.14^b^	22.89^a^	21.18^ab^	21.95^ab^	1.147	0.003	0.032	0.183	0.076	0.278
Serum AP, U/L	4087.9^bc^	4982.9^b^	1731.1^c^	1570.2^c^	3735.6^bc^	8142.7^a^	1486.1^c^	1332.5^c^	562.3	0.159	0.003	<0.0001	0.035	0.035

^a^ Abbreviations: OTM, olive tail moment; 8-OHdG, 8-hydroxy-2′-deoxyguanosine; MDA, malondialdehyde; Hsp70, Heat shock protein 70; AST, aspartate aminotransferase; ALT, alanine aminotransferase; GGT, Gamma-glutamyl transferase; AP, alkaline phosphatase.

^b^ Nomenclature of experimental groups as in Table 2.

^c^ Different superscript letters within the row show significant differences (p < 0.05). Mean values are based on 4 birds per replicate and three replicates per dietary treatment, n = 12.

### 3.4 Antioxidants and antioxidative enzymes levels

High n-3 PUFA inclusion decreased antioxidant levels, as measured by plasmatic α-tocopherol, liver vitamin E, plasmatic vitamin C, serum ACW and ACL, while SOD activity increased. In contrast, lower levels of antioxidants were measured under HS conditions compared to TN conditions, namely plasmatic α- and γ-tocopherol, liver vitamin E, plasmatic vitamin C, and serum ACW and ACL. Supplementation with additional antioxidants caused elevation in all measured parameters, with the exception of plasmatic γ-tocopherol and GPx activity in whole blood (Table 5). Significant interactions were observed between environment and antioxidant supplementation and dietary fat and antioxidant supplementation for plasma α-tocopherol (p = 0.0002 and p < 0.0001, respectively) and liver vitamin E (p = 0.010 and p = 0.001, respectively). The highest α-tocopherol and liver vitamin E levels were found when high antioxidants levels were paired with conventional fat inclusion under TN conditions, compared to low antioxidants and high n-3 PUFA levels under HS. In addition, a significant interaction was found between environment and antioxidant supplementation for ACL (p = 0.028) and between dietary fat and antioxidant supplementation for SOD (p = 0.027), with higher SOD activity measured when low antioxidants levels were administered with high PUFAs (Supplementary Table S4).

**TABLE 5:** Antioxidants and antioxidative enzymes levels measured in broiler plasma, serum and whole blood.

Item^a^/Diet^b^	Environment								p-Value					
	Thermoneutral (TN)				Heat stress (HS)				SEM	Environment (E)	Fat (F)	Antioxidants (A)	E × F	E × F × A
	NRC	NRC N-3	HAOX	HAOX N-3	NRC	NRC N-3	HAOX	HAOX N-3						
Plasma α-tocopherol, µg/mL	3.618^d^	2.698^d^	39.26^a^	25.75^bc^	3.653^d^	2.518^d^	28.19^b^	21.39^c^	1.382	0.0001	<0.0001	<0.0001	0.097	0.077
Plasma γ-tocopherol, µg/mL	1.822^ab^	2.246^a^	1.061^cd^	1.392^bcd^	1.644^abc^	1.404^bcd^	0.752^d^	0.849^d^	0.153	<0.0001	0.163	<0.0001	0.042	0.325
Liver vitamin E, µg/liver^c^	428.2^b^	318.2^b^	8127.3^a^	5562.1^a^	357.0^b^	149.4^b^	6325.6^a^	2728.9^b^	587.1	0.005	0.0002	<0.0001	0.499	0.576
Plasma vitamin C, µg/mL	11.16^d^	11.30^d^	17.14^ab^	15.54^abc^	14.48^bcd^	12.12^cd^	18.56^a^	15.43^abc^	0.896	0.024	0.004	<0.0001	0.091	0.678
Serum ACW, nmol/mL	333.3	325.6	419.8	355.7	435.0	328.1	410.0	361.5	26.79	0.191	0.004	0.104	0.274	0.135
Serum ACL, nmol/mL	162.3^c^	156.4^c^	244.7^ab^	231.3^ab^	231.7^ab^	190.2^bc^	271.8^a^	216.4^abc^	13.94	0.006	0.006	<0.0001	0.060	0.874
Whole blood SOD, U/gHGB	1243.5^abc^	1316.5^ab^	1044.0^c^	1087.7^c^	1132.0^bc^	1394.3^a^	1141.9^bc^	1123.4^bc^	47.74	0.467	0.011	<0.0001	0.356	0.071
Whole blood GPx, U/gHGB	185.6^b^	184.6^b^	373.0^a^	378.0^a^	179.2^b^	184.3^b^	341.9^a^	380.2^a^	11.95	0.294	0.164	<0.0001	0.248	0.423

^a^ Abbreviations: ACW, antioxidant capacity of water compounds; ACL, antioxidant capacity of lipid compounds; SOD, superoxide dismutase; GPx, Glutathione peroxidase; HGB, hemoglobin.

^b^ Nomenclature of experimental groups as in Table 2.

^c^ Calculated as vitamin E content in the liver.

^d^ Different superscript letters within the row show significant differences (p < 0.05). Mean values are based on 4 birds per replicate and three replicates per dietary treatment, n = 12.

## 4 Discussion

In the present trial, we evaluated the individual and combined effects of cyclic HS and the addition of dietary n-3 PUFAs on oxidative stress markers and antioxidative defense mechanisms. In addition, we aimed to investigate the effects of high levels of antioxidants under these conditions.

### 4.1 Growth performance

In this study, all three studied factors affected the performance parameters of the broilers. Cyclic HS lowered final BW and ADFI compared to TN conditions, n-3 PUFA inclusion worsened all three observed parameters compared to diets without n-3 PUFA, while high levels of antioxidants (HAOX groups) improved performance compared to NRC groups. Our results are in line with previously published research where exposure of broilers to HS has been reported to affect their growth performance, as evidenced by a decrease in final BW, BWG, FI and higher FCR (Sohail et al., 2012; Goo et al., 2019). However, no negative effects of high intake of n-3 PUFAs on broiler performance were found in similar trials (Leskovec et al., 2018; Pirman et al., 2021). Similar to our results, several studies showed a protective effect of the combination of vitamins E and C (Attia et al., 2017), and vitamin E and Se (Habibian et al., 2016) on the growth parameters of broilers under HS conditions. We confirmed that in case of high n-3 PUFA intake or cyclic HS, additional supplementation of antioxidants should be considered to maintain optimal broiler performance.

### 4.2 Blood chemistry parameters and serum electrolytes

In the present study, exposure of broilers to HS showed decreased levels of TCO_2_, HCO_3_-, BEecf and Na+ and increased levels of s O_2_, Glu and Cl- compared to birds reared under TN conditions. As for blood pH and pCO_2_, the parameters indicated that birds reared under HS conditions suffered from respiratory alkalosis compared to birds reared under TN conditions. On the other hand, no differences were found between groups in blood levels of sO_2_, Hct and HGB in relation to environmental conditions. Our results are consistent with previous studies in which exposure of broilers to acute HS increased blood pH and Glu levels and decreased blood pCO_2_, TCO_2_, HCO_3_-, iCa and Na+ (Shakeri et al., 2019; Beckford et al., 2020). Moreover, blood iCa decreased in the NRC N-3 group under HS, exerting negative synergistic effects. In contrast, supplementation with supranutritional antioxidants increased blood iCa concentrations. We can conclude that HS caused typical physiological response, while the additional dietary stress with high n-3 PUFAs and supranutritional antioxidants had only minor effects on the measured physiological parameters.

### 4.3 Markers of oxidative stress in blood

Oxidative damage to DNA and lipids is a good indicator of oxidative stress *in vivo*. Although oxidation products of n-3 PUFAs are involved in oxidative DNA damage, a high intake of linseed oil in this study did not lead to increased DNA damage in blood lymphocytes of broiler chickens measured with the comet assay. In contrast, previous studies reported that the inclusion of high levels of linseed oil in the diet of broilers (Voljč et al., 2013) and pigs (Frankič and Salobir, 2011; Leskovec et al., 2019) was associated with increased DNA damage, which was mitigated by vitamin E supplementation that reduced DNA fragmentation of lymphocytes. Contrary, our results indicated that HS increased DNA damage in broiler lymphocytes, as measured by the comet assay. Serum levels of 8-OHdG, an important biomarker of oxidative DNA damage, were also higher in the HAOX groups than in the NRC groups and in the broilers exposed to HS Although higher DNA damage was expected under HS, the increased 8-OHdG levels in birds receiving antioxidant supplementation should be interpreted with caution. Higher antioxidant levels could either reflect increased oxidative DNA damage or contribute to a higher rate of DNA repair, which in some cases increases the levels of oxidative markers (Ferguson et al., 2006). It appears that the DNA damage was mostly affected by HS, while the additional antioxidant supplementation may have contributed to higher 8-OHdG levels, probably indicating an accelerated DNA repair rate rather than increased damage. F2-isoprostanes and MDA are commonly used markers for the quantification of lipid peroxidation. In the present study, plasma F2-isoprostane levels were lower in both groups supplemented with linseed oil, probably due to lower arachidonic acid content in tissues after long-term linseed oil supplementation (data not shown). This result is in contrast to previous studies in pigs (Frankič and Salobir, 2011) and broilers (Pirman et al., 2021), in which no changes in F2-isoprostane levels were observed with a diet high in n-3 PUFAs. In addition, plasma F2-isoprostane levels were lowest in the HAOX-N-3 group, suggesting a protective antioxidant effect and consistent with the results in pigs in which vitamin E lowered F2-isoprostane levels (Leskovec et al., 2019). No differences were found in plasma or liver MDA concentrations between TN and HS environments. However, previous studies have shown increased lipid peroxidation under HS, with elevated MDA levels in skeletal muscle (Ghazi Harsini et al., 2012), breast meat (Habibian et al., 2016), liver and serum (Yang et al., 2010) of broilers. In addition, our results showed higher MDA concentrations in plasma and liver in the NRC N-3 group than in the other experimental groups, which is consistent with previous findings of increased MDA concentrations in broilers fed n-3 PUFA-enriched diets (Voljč et al., 2011; Voljč et al., 2013). These results emphasize the importance of additional antioxidant supplementation to counteract oxidative damage caused by high n-3 PUFA levels and HS. The observed significant negative interaction between cyclic HS and high n-3 PUFA intake, as reflected in liver MDA levels, emphasizes the combined deleterious effect of both stressors on lipid oxidation. Notably, antioxidants such as vitamin E (Khan et al., 2011; Voljč et al., 2011), vitamin C (Sahin et al., 2002; Jena et al., 2013) and selenium (Kumbhar et al., 2018) have been shown to reduce MDA levels and prevent lipid peroxidation under both dietary and HS conditions. The results of this study suggest that high intake of n-3 PUFAs and cyclic HS independently cause oxidative damage to DNA and lipids, with a notable interaction affecting lipid oxidation in the liver. These results emphasize the importance of additional antioxidant supplementation to counteract oxidative damage caused by n-3 PUFA and heat stress. CORT serves as a stress indicator and can reflect increased inflammatory processes in broilers. In this study, exposure to HS significantly increased serum CORT levels in broilers, which is consistent with previous findings (Najafi et al., 2015; Ramiah et al., 2019). While no interaction was observed between the dietary treatments and environmental conditions, the combination of HS and high n-3 PUFA intake further increased CORT levels. In contrast to the findings of Tari et al. (2019), enrichment with n-3 PUFAs under HS did not decrease CORT levels. Although it has been suggested that antioxidants such as vitamin C reduce CORT secretion (Saiz del Barrio et al., 2020), this effect could not be confirmed in our results. Plasma concentrations of heat shock protein 70 (Hsp70) did not increase after HS exposure. This could be due to acclimation, where birds adapt to the heat over time, resulting in no further increase in Hsp70 concentrations after the initial increase in Hsp70 expression (Mahmoud and Edens, 2005). Previous studies (Seifi et al., 2018; Tari et al., 2019) reported increased Hsp70 gene expression in broilers fed diets high in n-3 PUFAs under HS, but this response was not observed in the current study. However, antioxidant supplementation as demonstrated by Kurashova et al. (2020), may have alleviated oxidative stress and reduced Hsp70 expression during prolonged HS exposure. Although dietary antioxidants had no direct effect on plasma Hsp70 concentrations in this study, supplementation with vitamins E, C and organic selenium appeared to reduce Hsp70 expression, highlighting their potential role in alleviating oxidative damage caused by HS (Mahmoud and Edens, 2005; Jang et al., 2014; Calik et al., 2022a). In conclusion, exposure to HS increased serum CORT levels, and the combined effects of external stressors such as high n-3 PUFAs and HS should be further investigated to confirm their negative joint effects. Liver enzymes in serum are commonly used as diagnostic markers to assess liver damage. In this study, no differences in ALT activity were found between dietary treatments or environmental conditions, which is noteworthy since ALT is more specific for liver injury than AST. However, AST levels were higher in the HAOX group than in the NRC group. In addition, the combination of HS and high n-3 PUFA intake, especially in the NRC-N-3 group, resulted in increased GGT and AP activities, suggesting a synergistic negative effect on liver function. Antioxidant supplementation showed a protective effect by reducing AP activity compared to the non-supplemented groups. This is consistent with previous studies showing that linseed oil increased plasma ALT and GGT levels in pigs, but these effects were attenuated by antioxidants (Frankič and Salobir, 2011; Leskovec et al., 2019). Similarly, previous studies have reported increased AST activity under HS conditions, which was attenuated by supplementation with vitamins E and C, while ALT activity remained unaffected (Attia et al., 2017; Tang et al., 2022).

### 4.4 Antioxidants and antioxidative enzymes levels

Consistent with previous studies, α-tocopherol supplementation (HAOX groups) significantly increased plasma α-tocopherol levels in all supplemented groups (Konieczka et al., 2015; Leskovec et al., 2018). These levels were higher under TN conditions than under HS conditions, suggesting increased utilization of vitamin E during HS. The observed negative correlation between α-tocopherol and γ-tocopherol levels is likely due to competitive binding, which is consistent with the results of our previous studies (Voljč et al., 2011; Leskovec et al., 2018). In addition, liver vitamin E levels were lower in the HAOX N-3 group under HS, suggesting a faster depletion of vitamin E under combined stressors, which in turn suggests a higher dietary vitamin E requirement under stressful conditions. Dietary antioxidants also increased plasma vitamin C levels, with higher concentrations in the HAOX groups than in the HAOX N-3 groups, possibly reflecting increased utilization of vitamin C under dietary stress. This observation is supported by Ghazi et al. (2015), who reported increased serum vitamin C levels in broilers supplemented with ascorbic acid under HS. The present results suggest that endogenous vitamin C synthesis may not be sufficient to counteract lipid oxidation, thus requiring external supplementation. Interestingly, in the present study, plasma vitamin C concentrations were higher under HS than under TN, which may reflect increased *in vivo* synthesis of vitamin C in response to HS. High linseed oil intake (NRC N-3 group) decreased serum ACW levels compared to the antioxidant-supplemented groups, suggesting that dietary vitamin C plays a role in maintaining homeostasis and physiological function of birds during oxidative stress associated with high n-3 PUFA intake. Increased plasma vitamin C levels in the antioxidant-supplemented groups suggest that other endogenous water-soluble antioxidants may be preserved or replaced by increased vitamin C activity. Similarly, linseed oil supplementation reduced ACL levels in the NRC N-3 group. However, serum ACL levels increased under HS, likely due to increased endogenous antioxidant synthesis. The observed increase in ACL levels in the HAOX groups is consistent with the effects of vitamin E supplementation and its restoration by vitamin C (Sahin et al., 2002; Leskovec et al., 2018). A significant interaction between the dietary treatments and the rearing environment showed higher serum ACL levels in the NRC group under HS than under TN, indicating a stress-induced response through increased synthesis of endogenous antioxidants. SOD activity was increased in the both NRC N-3 groups compared to all other groups, probably in response to the oxidative stress induced by the high intake of n-3 PUFAs. Antioxidant supplementation reduced SOD activity compared to the NRC N-3 groups, suggesting a regulatory balance between endogenous and dietary antioxidants. This finding aligns with those of Kostadinović et al. (2016), who reported increased SOD activity in broilers fed extruded flaxseed. Conversely, Delles et al. (2014) reported that antioxidants restored SOD activity suppressed by highly oxidized diets. Similarly, previous studies have shown that supplementation with vitamin E, vitamin C or folic acid, either alone or in combination, improved antioxidant status and increased SOD activity in broilers under HS (Jena et al., 2013; Gouda et al., 2020). Moreover, GPx activity was increased in all HAOX groups, suggesting that dietary selenium supplementation was effective in increasing GPx activity, which is consistent with previous studies (Kumbhar et al., 2018; Woods et al., 2021). Furthermore, HS had no effect on GPx activity, confirming the findings of Woods et al. (2021).

## 5 Conclusion

The results of the present study indicate that the oxidative status and antioxidative defense mechanisms of broilers were negatively affected by high n-3 PUFA intake and cyclic HS, with evidence of their combined detrimental effects. High n-3 PUFA intake induced lipid oxidation, reflected by increased F2-isoprostane and MDA levels in plasma and liver, and disrupted antioxidative defense by decreasing antioxidant levels while increasing SOD activity, contributing to oxidative stress. Cyclic HS triggered typical physiological responses, including respiratory alkalosis characterized by changes in serum electrolytes, enhanced oxidative damage by increasing DNA fragmentation and serum CORT, and disrupting antioxidative defense mechanisms. Combined exposure to both stressors further exacerbated oxidative stress as measured by higher liver MDA levels, lower plasma γ-tocopherol levels and higher serum AP levels, suggesting further detrimental effects on antioxidative defense compared to separate exposure to the stressors. According to our results, the first hypothesis was partially confirmed, as only a few parameters related to oxidative stress and antioxidative defense were negatively affected by the combined stressors.

Additional supplementation with vitamins E, C and selenium improved growth performance, reduced lipid peroxidation and serum CORT levels and improved antioxidant protection as elevated plasma α-tocopherol and vitamin C concentrations, serum ACL levels, whole blood GPx activity and reduced AP levels. While antioxidant supplementation should be considered to mitigate the negative effects of dietary- and heat-induced oxidative stress, it may not be necessary under stress-free conditions. Thus, our second hypothesis was confirmed, demonstrating clear benefit of antioxidant supplementation under high oxidative load but not under stress-free conditions. However, their role in boosting antioxidative reserves and reducing potential oxidative damage cannot be dismissed. In conclusion, high n-3 PUFA intake and HS, both individually and in combination, induce oxidative stress in broilers, which could be partially mitigated by additional supplementation with vitamins E, C and selenium. Further studies are needed to determine the appropriate levels of antioxidants under different stress conditions to maximize their protective effect.

## Data Availability

The original contributions presented in the study are included in the article/Supplementary Material, further inquiries can be directed to the corresponding author.
