# Supplementary material for: Interactive effects of high n-3 PUFA intake and cyclic heat stress under two dietary antioxidant levels in broiler chickens
Source: Front Physiol. 2025 Apr 24;16:1594095. doi: 10.3389/fphys.2025.1594095 (PMC12058473; doi:10.3389/fphys.2025.1594095)
Supplement: Supplementary file 1 [file Table1.docx]

Supplementary Material

Interactive effects of high n-3 PUFA intake and cyclic heat stress under two dietary antioxidant levels in broiler chickens

Vida Rezar^1*^, Manca Pečjak Pal^1^, Alenka Levart^1^, Alenka Nemec Svete^2^, Tatjana Pirman^1^, Janez Salobir^1^, Jakob Leskovec^1,3^

^1^Department of Animal Science, Biotechnical Faculty, University of Ljubljana, Ljubljana, Slovenia

^2^Clinic for Surgery and Small Animal Medicine, Veterinary Faculty, University of Ljubljana, Ljubljana, Slovenia

^3^Animal Nutrition, Institute for Food and Agricultural Research and Technology (IRTA), Tarragona, Spain

*** Correspondence:** vida.rezar@bf.uni-lj.si

**Table S1:** Analysis of variance table with probabilities for environmental conditions and dietary treatments (dietary fat treatments and antioxidant supplementation) for the variables of growth performance

|  | *p > f* | | | |
| --- | --- | --- | --- | --- |
| Effect | BW_21 d | BW_40 d | ADFI 21-40 d | FCR_21-40 d |
| E × A | 0.750 | 0.916 | 0.447 | 0.381 |
| F × A | 0.220 | 0.010 | 0.114 | 0.058 |

BW = Body weight; ADFI = Average feed intake; FCR = Feed conversion ratio; E = environmental conditions; F = dietary fat treatments; A = antioxidant supplementation.

**Table S2:** Analysis of variance table with probabilities for environmental conditions and dietary treatments (dietary fat treatments and antioxidant supplementation) for the variables of blood chemistry parameters in venous blood and serum electrolyte (Na^+^, K^+^, and Cl^-^) levels

|  | *p > f* | | | | | | | | | | | | | |
| --- | --- | --- | --- | --- | --- | --- | --- | --- | --- | --- | --- | --- | --- | --- |
| Effect | TCO_2_ | pCO_2_ | pO_2_ | sO_2_ | pH | HCO_3_^-^ | BEecf | Glu | Hct | HGB | Na^+^ | K^+^ | Cl^-^ | iCa |
| E × A | 0.242 | 0.684 | 0.197 | 0.290 | 0.373 | 0.200 | 0.049 | 0.635 | 0.934 | 0.960 | <0.0001 | 0.689 | 0.105 | 0.003 |
| F × A | 0.824 | 0.678 | 0.826 | 0.243 | 0.460 | 0.823 | 0.832 | 0.021 | 0.677 | 0.672 | 0.201 | 0.285 | 0.265 | 0.0004 |

TCO_2_ = Total CO_2_ concentration; pCO_2_ = Partial pressure of CO_2_; pO_2_ = Partial pressure of O_2_; sO_2_ = Oxygen saturation; HCO_3_^-^ = Bicarbonate; BEecf = Base excess of the extracellular fluid; Glu = Blood glucose; Hct = Hematocrit; HGB = Hemoglobin; iCa = Ionized calcium; VB = Venous blood; E = environmental conditions; F = dietary fat treatments; A = antioxidant supplementation.

**Table S3:** Analysis of variance table with probabilities for environmental conditions and dietary treatments (dietary fat treatments and antioxidant supplementation) for the variables of oxidative stress parameters, corticosterone (CORT) and heat shock protein 70 (Hsp70) levels, and activities of liver enzymes

|  | *p > f* | | | | | | | | | | | |
| --- | --- | --- | --- | --- | --- | --- | --- | --- | --- | --- | --- | --- |
| Effect | Tail DNA | OTM | 8-OHdG | F2-isoprostanes | Plasma MDA | Liver MDA | CORT | Hsp70 | AST | ALT | GGT | AP |
| E × A | 0.922 | 0.164 | 0.178 | 0.610 | 0.072 | 0.562 | 0.291 | 0.575 | 0.561 | 0.540 | 0.578 | 0.048 |
| F × A | 0.965 | 0.827 | 0.188 | 0.163 | <0.0001 | 0.013 | 0.877 | 0.461 | 0.933 | 0.718 | 0.054 | 0.001 |

OTM = Olive Tail Moment; 8-OHdG = 8-hydroxy-2'-deoxyguanosine; MDA = Malondialdehyde; CORT = Corticosterone; Hsp70 = Heat shock protein 70; AST = Aspartate aminotransferase; ALT = Alanine aminotransferase; GGT = Gamma-glutamyl transferase; AP = Alkaline phosphatase; E = environmental conditions; F = dietary fat treatments; A = antioxidant supplementation.

**Table S4:** Analysis of variance table with probabilities for environmental conditions and dietary treatments (dietary fat treatments and antioxidant supplementation) for the variables of antioxidants and antioxidative enzymes

|  | *p > f* | | | | | | | |
| --- | --- | --- | --- | --- | --- | --- | --- | --- |
| Effect | Plasma α-Toc | Plasma γ-Toc | Liver vitamin E | Plasma vitamin C | ACW | ACL | SOD | GPx |
| E × A | 0.0002 | 0.701 | 0.010 | 0.233 | 0.158 | 0.028 | 0.226 | 0.514 |
| F × A | <0.0001 | 0.577 | 0.001 | 0.291 | 0.979 | 0.599 | 0.027 | 0.251 |

Toc= Tocopherol; ACW = Antioxidant capacity of water compounds; ACL = Antioxidant capacity of lipid compounds; SOD = Superoxide dismutase; GPx = Glutathione peroxidase; HGB = Hemoglobin E = environmental conditions; F = dietary fat treatments; A = antioxidant supplementation.
